# The effects of antiepileptic drugs on the growth of glioblastoma cell lines

**DOI:** 10.1007/s11060-016-2056-6

**Published:** 2016-01-13

**Authors:** Ching-Yi Lee, Hung-Yi Lai, Angela Chiu, She-Hung Chan, Ling-Ping Hsiao, Shih-Tseng Lee

**Affiliations:** Department of Neurosurgery, Chang-Gung Memorial Hospital, Chang-Gung University College of Medicine, 5 Fu-Shing Street, 333, Kweishan, Taoyuan, Taiwan

**Keywords:** Glioblastoma, Antiepileptic, Oxcarbazepine, Valproic acid, Temozolomide

## Abstract

To determine the effects of antiepileptic drug compounds on glioblastoma cellular growth, we exposed glioblastoma cell lines to select antiepileptic drugs. The effects of selected antiepileptic drugs on glioblastoma cells were measured by MTT assay. For compounds showing significant inhibition, cell cycle analysis was performed. Statistical analysis was performed using SPSS. The antiepileptic compounds selected for screening included carbamazepine, ethosuximide, gabapentin, lamotrigine, levetiracetam, magnesium sulfate, oxcarbazepine, phenytoin, primidone, tiagabine, topiramate, valproic acid, and vigabatrin. Dexamethasone and temozolomide were used as a negative and positive control respectively. Our results showed temozolomide and oxcarbazepine significantly inhibited glioblastoma cell growth and reached IC_50_ at therapeutic concentrations. The other antiepileptic drugs screened were unable to reach IC_50_ at therapeutic concentrations. The metabolites of oxcarbazepine were also unable to reach IC_50._ Dexamethasone, ethosuximide, levetiracetam, and vigabatrin showed some growth enhancement though they did not reach statistical significance. The growth enhancement effects of ethosuximide, levetiracetam, and vigabatrin found in the study may indicate that these compounds should not be used for prophylaxis or short term treatment of epilepsy in glioblastoma. While valproic acid and oxcarbazepine were effective, the required dose of valproic acid was far above that used for the treatment of epilepsy and the metabolites of oxcarbazepine failed to reach significant growth inhibition ruling out the use of oral oxcarbazepine or valproic acid as monotherapy in glioblastoma. The possibility of using these compounds as local treatment is a future area of study.

## Introduction

Gliomas represent approximately 31 % of all primary brain and central nervous system (CNS) tumors with glioblastoma multiforme (GBM) accounting for almost 17 % of all tumors [1] Long term survivors of GBM are rare with a median survival of less than 1 year being typical and a 5 year survival rate of less than 5 % [1–5].

Treatment of high grade gliomas typically includes surgical excision followed by chemotherapy and symptom management. GBM is treated with temozolomide (TMZ) given concomitantly with radiation therapy (RT) to increase efficacy and then continued as adjuvant treatment for 6 to 12 months afterwards [4, 6–14].

Epileptic seizures are the presenting symptom of intracranial lesions 30–50 % of the time [15, 16]. 10–30 % of patients who are seizure free at diagnosis develop seizures at some point throughout their disease progression [5, 16, 17] Patients that develop seizures are treated with anti-epileptic drugs (AEDs) and some physicians may use AEDs prophylactically to prevent possible seizure complications after the diagnosis of cranial lesions or after surgery. Since the pathophysiology behind these seizures is complex, many patients are likely to require multiple AEDs in combination.

Valproic acid (VPA) is used for the treatment tumor associated epilepsy (TAE) due to studies showing VPA to have anticancer properties [4, 5, 9, 11, 12, 15–24]. In our previous study to examine the correlation between the use of VPA in patients diagnosed with GBM and patient survival, the results concluded that VPA, at serum concentrations used for the treatment of seizures (50–100 ug/ml), may be beneficial and lead to a better prognosis in the treatment of GBM when combined with total resection of tumors and post-operative chemotherapy [25]. While some studies examine VPA as a possible anti-cancer agent and others examine the efficacy of AEDs for seizure reduction in glioma and GBM, few look at the direct possible anti-cancer effects of AEDs.

This cell culture study aims to address the following points: 1) if VPA alone at the clinical therapeutic level used for epilepsy treatment in patients has equivalent anti-cancer effects in cell cultures, 2) at what concentration does VPA alone demonstrate anti-cancer effects, and 3) what other AEDs influence the growth of glioma/GBM cells when used at the targeted therapeutic levels for seizure control. Identifying the optimal use of VPA and determining the effects of other AEDs on glioma/GBM cells will allow for enhanced therapy which is important in patients with GBM who have poor survival even when given optimal therapy.

## Materials and methods

### Cell line selection and cell culture

Human glioma cell lines U-87 MG and T98G [American type culture collection (ATCC), Manassas, VA] were used in this study. The cell line U87 MG, used in multiple studies, represents typical glioblastoma [26–28] The T98 cell line represents resistant glioblastoma due to TMZ resistance [29] Cells were cultured in Eagle’s minimal essential medium (EMEM) supplemented with 10 % Fetal bovine serum (FBS), and 1 % antibiotic antimycotic solution and placed in a standard humidified incubator at 37 °C and 5 % CO_2_/95 % air atmosphere.

### Drug compound selection

Drugs compounds were chosen based on the antiepileptic drugs available on the Chang Gung Memorial Hospital formulary. The compounds used are listed in Table 1. Temozolomide acts as a positive control showing growth inhibition. Dexamethasone (DEX) acts as a negative control to show growth enhancement in GBM [30, 31] Licarbazepine (R-(−)-10-hydroxy- 10,11-dihydro- carbamazepine or monohydroxycarbamazepine (R-(−)-MHC)) and eslicarbazepine (S-(+)-10-hydroxy- 10,11-dihydro- carbamazepine or monohydroxycarbamazepine (S-(+)-MHC)), metabolites of the prodrug OXC, were added to the compounds selected after preliminary data indicated that OXC induced significant growth inhibition.

**Table 1:** List of antiepileptic compounds used in this study and associated abbreviations, therapeutic plasma levels for epilepsy treatment, toxicity levels, and experimental dosing concentrations

Drug name	Drug abbreviation	Therapeutic μg/ml	Toxic	Dosing concentrations μg/ml
Temozolomide	TMZ	4–11	Determined by myelosupression/hepatotoxicity	0.04, 0.4, 4, 10, 20, 40
Dexamethasone	DEX	2.6–18	Rat LD_50_ 3 gm/kg	0.008, 0.08, 0.8, 16, 40
Valproic acid	VPA	50–150	450–850 μg/ml	10, 100, 250, 500, 1000
Carbamazepine	CBZ	4–12	18–42.8 μg/ml	1, 2, 4, 8, 16
Ethosuximide	ESX	40–160	>150 μg/ml	10, 20, 40, 80, 160
Gabapentin	GBP	2–8.6	40–100 g	0.2, 2, 10, 20
Lamotrigine	LTG	0.5–5.4	13–62.4 μg/ml	0.2, 2, 10, 20
Levetiracetam	LEV	6.85–72	60–400 μg/ml	5, 10, 20, 40, 80
Magnesium sulfate	MgSO_4_	240.83–421.45	Plasma Mg level of 4–12 mEq/L	5.55, 16.6, 50, 150, 450
Oxcarbazepine	OXC	3–35	30–40 μg/ml	2.5, 5, 10, 20, 40
Licarbazepine (R-(−)-10-hydroxy-10,11-dihydro-carbamazepine/monohydroxycarbamazepine)	R-(−)-MHC	3–35	30–40 μg/ml	12.5, 25, 75
Eslicarbazepine (S-(+)-10-hydroxy- 10,11-dihydro- carbamazepine/monohydroxycarbamazepine)	S-(+)-MHC	3–35	30–40 μg/ml	12.5, 25, 75
Phenytoin	PHT	10–20	20–40 μg/ml	0.04, 0.4, 4, 20, 40
Primidone	PRM	5–12	40–80 μg/ml	0.2, 1, 5, 12.5, 25
Tiagabine	TGB	0.04–0.55	0.7–4.6 μg/ml	0.0008, 0.008, 0.08, 0.8
Topiramate	TPM	2–19	9.4–170 μg/ml	0.2, 2, 10, 20
Vigabatrin	VBT	5.3	60 g	0.04, 0.4, 4, 20, 40

### Reagents

EMEM powder and antibiotic/antimycotic solution were obtained from Gibco, Grand Island, NY. FBS and phosphate buffered saline (PBS) were obtained from Hyclone through Thermo Scientific. Dimethyl sulfoxide (DMSO), ethanol (99 %) (EtOH), TMZ, DEX, VPA, carbamazepine (CBZ), ethosuximide (ESX), gabapentin (GBP), lamotrigine (LTG), levetiracetam (LEV), magnesium sulfate (MgSO_4_), oxcarbazepine (OXC), phenytoin (5, 5 diphenylhydantoin) (PHT), primidone (PRM), tiagabine (TGB), topiramate (TPM), and vigabatrin (VBT) were obtained from Sigma-Aldrich, St. Louis, MO. R-(−)-MHC and S-(+)-MHC were obtained from Santa Cruz, Dallas, TX.

### Dose determination

The drug concentration ranges chosen for this study were taken from estimated drug plasma levels reported to be found in patients with adjustments for protein binding as needed [32–35]. The range was based on the minimum reported effective plasma level and the maximum reported level found in patients [35–38]. Toxic plasma levels were determined from Micromedex and other reports [35–38]. The drug concentration range for VPA was extended to toxic levels to ensure that IC_50_ for VPA would be obtained due to reports of VPA as an anti-cancer agent (Table 1).

### Experimental procedure

Cells were collected and seeded in 24-well plates at a density of 1 × 10^4^ cells/well (U-87 MG) and 5 × 10^3^ cells/well (T98G) after optimization at the beginning of the experiments. After 24 h, drug was added to each well to reach the dosing concentrations listed in Table 1. The treated cells were incubated for 72 h after which cell viability was assessed.

### Cytotoxicity/cell viability assay

Cytotoxicity/cell viability analysis was performed using the 3-(4, 5-dimethylthylthiazol-2-yl)-2, 5-diphenyltetrazolium bromide (MTT) method. 50 µl of MTT tetrazolium salt (Sigma) dissolved in PBS at a concentration of 5 mg/ml was added to each well at 70 h post treatment and incubated for 2 h. The medium was then aspirated from each well and 200 µl of DMSO was added to dissolve the formazan crystals. 150 µl of the resulting solution was transferred to a 96-well plate and the absorbance of each well was obtained using a Tecan infinite M200 Pro plate reader at a wavelength of 570 nm. Each experiment was replicated multiple times with similar results.

### Cell cycle analysis

If growth inhibition reached 50 % (IC_50_), cell cycle analysis was performed. Cells were trypsinized and harvested and fixed in 70 % ethanol for at least 30 min at 4 °C. After the removal of the alcoholic fixative, they were stained with a solution containing 50 µg/ml Propidium Iodide (PI) and 100 µg/ml RNase A (Sigma) for approximately 30 min at room temperature while being protected from light. Samples were then measured using flow cytometry (FACScans, Becton–Dickinson). Ten thousand events per sample were acquired and the data was analyzed using Cell Quest software (Becton–Dickinson).

### Statistical analysis

The data were expressed as mean ± standard deviation (SD). Statistical analysis was performed with Statistical Product and Service Solutions (SPSS) using analysis of variance (ANOVA). The criterion for statistical significance was taken as p < 0.05.

## Results

### Cytotoxicity/cell viability

**Fig. 1:**
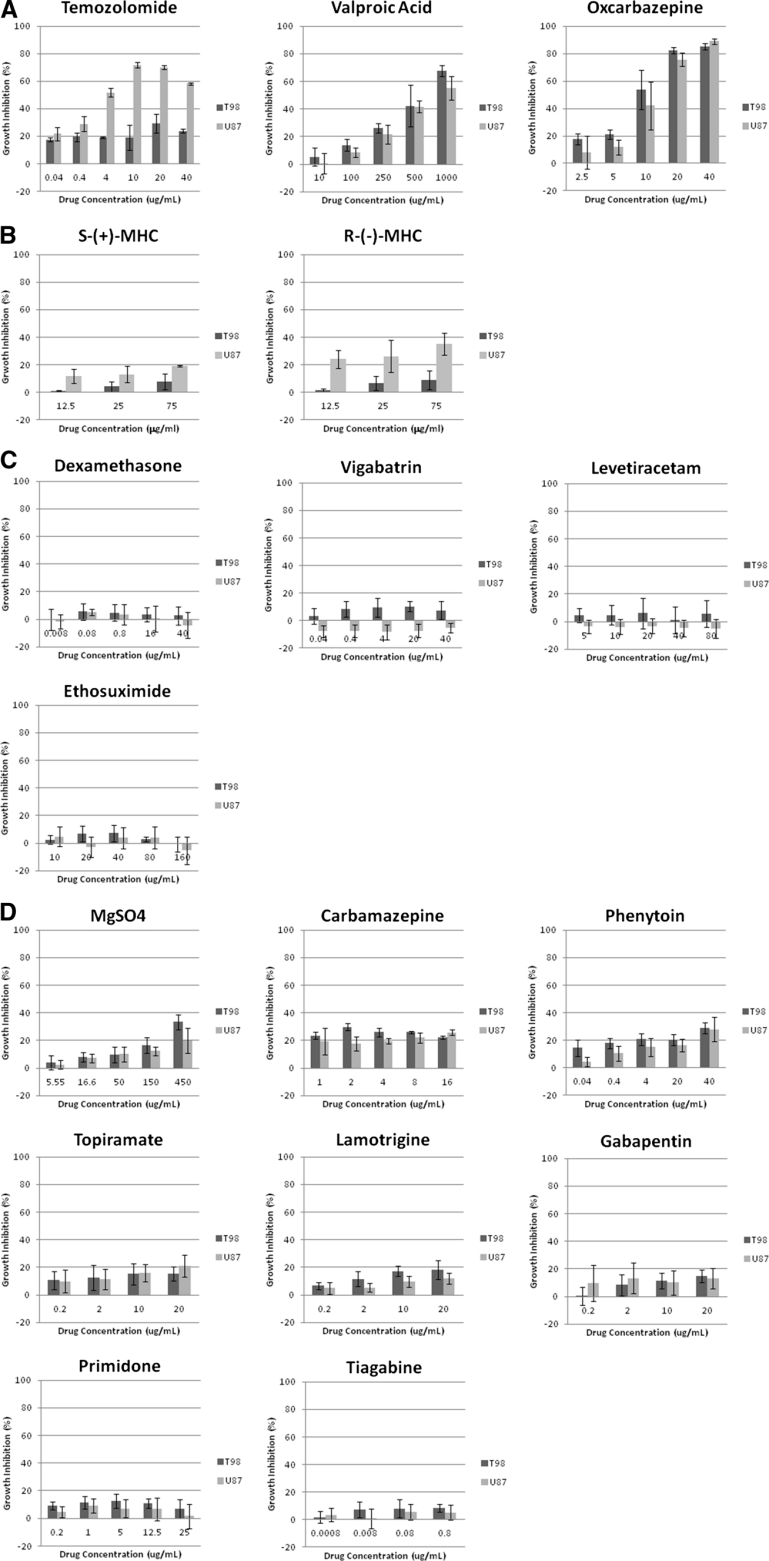
Growth inhibition for all compounds and cell lines. **a** Growth inhibition for TMZ, VPA, and OXC, the three compounds that attained over 50 % growth inhibition. **b** Growth inhibition for the OXC metabolites R-(−MHC and S-(+)-MHC. **c** Growth inhibition and enhancement for DEX, VBT, LEV, and ESX, the four compounds that displayed both growth inhibition and growth enhancement. **d** Growth inhibition for MgSO_4_, CBZ, OHT, TPM, LTG, GBP, PRM, and TGB, the compounds that showed growth inhibition, but did not attain over 50 % growth inhibition

**Fig. 2:**
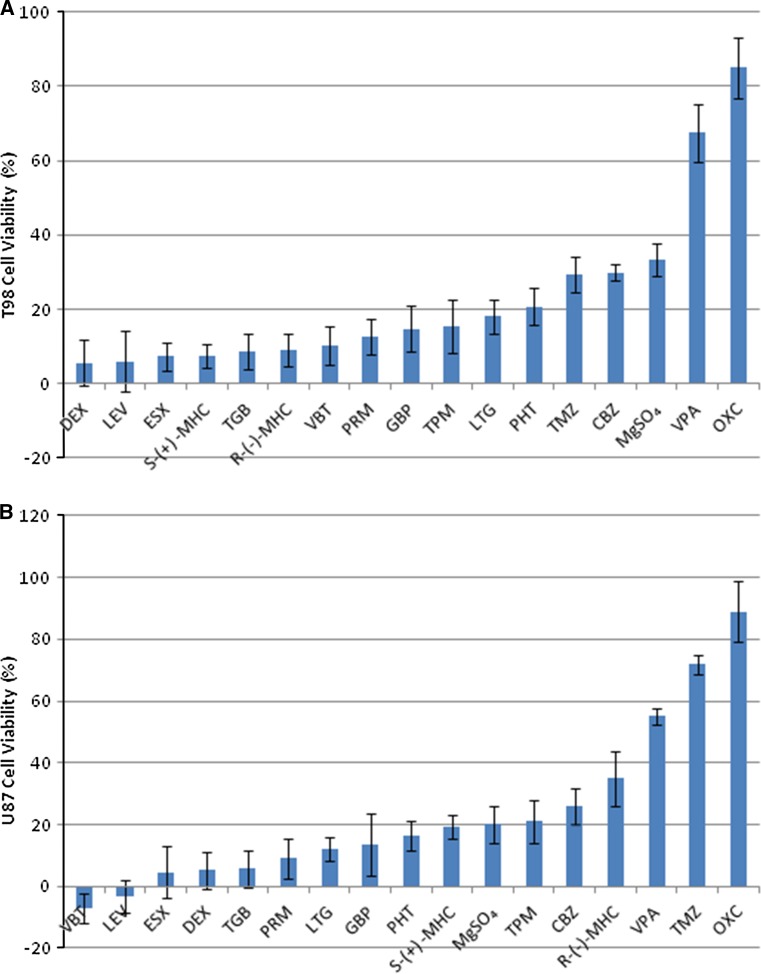
**a** The 17 compounds ordered according to maximum percent of growth inhibition after treatment using the T98 cell line with a minimum of three replicates. **b** The 17 compounds ordered according to maximum percent of growth inhibition after treatment using the U87 cell line with a minimum of three replicates

Overall growth inhibition for all compounds and cell lines are shown in Fig. 1. Figure 2 depicts the maximum growth inhibition reached by each compound and can be separated into control compounds, non-effective compounds, and effective compounds, with effective compounds consisting of those that reached half maximal inhibitory concentration (IC_50_) as shown in Table 2.

**Table 2:** IC_50_ of the effective compounds and compound metabolites screened with the U87 and T98 cell lines

IC_50_ drug concentrations			
	Drug name	U87 μg/ml	T98
AED	Oxcarbazepine	12.35	9.45 μg/ml
AED	Valproic acid	808.82	652.78 μg/ml
Positive CTL	Temozolomide	3.4	*

* IC_50_ was not reached in the T98 cell line using the highest drug concentration (40 μg/ml) of TMZ

### Control group

For TMZ, the growth inhibition for the T98G cell line (means and SDs) for each concentration was 17.6 ± 1.5 % (0.04 μg/ml), 19.4 ± 3.1 % (0.4 μg/ml), 19.2 ± 0.7 % (4 μg/ml), 18.9 ± 9.1 % (10 μg/ml), 29.3 ± 6.8 % (20 μg/ml), and 23.8 ± 1.4 % (40 μg/ml). For U-87 MG it was 21.8 ± 4.8 % (0.04 μg/ml), 29.0 ± 5.5 % (0.4 μg/ml), 51.6 ± 3.1 % (4 μg/ml), 71.8 ± 2.2 % (10 μg/ml), 70.0 ± 1.4 % (20 μg/ml), and 58.2 ± 0.8 % (40 μg/ml). The T98 cell line was unable to reach IC_50_ at the concentrations used and only the 20 μg/ml concentration showed statistically significant growth inhibition (p value = 0.01). For U87, statistical significance was found at all concentrations (p values of 0.012 (0.4 μg/ml) and <0.001 (4,10, 20, 40 μg/ml)) and IC_50_ was calculated to be 3.4 μg/ml.

For DEX, the growth inhibition for the T98G cell line for each concentration was −0.3 ± 7.5 % (0.008 μg/ml), 5.6 ± 6.0 % (0.08 μg/ml), 4.8 ± 5.8 % (0.8 μg/ml), 3.5 ± 5.0 % (16 μg/ml), and 2.7 ± 6.5 % (40 μg/ml). For U-87 MG it was −1.7 ± 5.1 % (0.008 μg/ml), 5.3 ± 2.4 % (0.08 μg/ml), 3.5 ± 7.4 % (0.8 μg/ml), 0.3 ± 9.2 % (16 μg/ml), and −4.2 ± 9.6 % (40 μg/ml). While some concentrations resulted in a negative growth inhibition, it did not reach statistical significance.

### Non-effective group

The growth inhibition for R-(−)-MHC in the T98G cell line (means and SDs) for each concentration was 1.3 ± 1.2 % (13 μg/ml), 6.5 ± 5.3 % (25 μg/ml), and 8.9 ± 6.7 % (76 μg/ml). For U-87 MG it was 24.0 ± 6.8 % (13 μg/ml), 26.2 ± 11.7 % (25 μg/ml), and 34.9 ± 8.0 % (76 μg/ml). The growth inhibition for S-(+)-MHC in the T98G cell line for each concentration was 1.2 ± 0.4 % (13 μg/ml), 4.2 ± 3.6 % (25 μg/ml), and 7.6 ± 5.6 % (76 μg/ml). For U-87 MG it was 11.6 ± 5.2 % (13 μg/ml), 13.0 ± 6.0 % (25 μg/ml), and 19.3 ± 0.7 % (76 μg/ml).

LTG, MgSO_4_, and PHT showed statistically significant growth inhibition in both cell lines, however, they did not reach IC_50_. Exposure to CBZ, ESX, and GBP resulted in statistically significant growth inhibition in the T98G cell line only, but also failed to reach IC_50_. The other antiepileptics, LEV, PRM, TGB, TPM, and VBT, used in this study failed to reach statistically significant growth inhibition. While VBT, LEV, ESX, and DEX did show some growth enhancement in the U87 and T98 cell lines, it was not statistically significant level.

### Effective group

For VPA, the growth inhibition for the T98G cell line (means and SDs) for each concentration was 5.2 ± 4.1 % (10 μg/ml), 13.9 ± 4.8 % (100 μg/ml), 26.0 ± 5.3 % (250 μg/ml), 42.3 ± 11.7 % (500 μg/ml), and 67.5 ± 3.7 % (1000 μg/ml). For U-87 MG it was 0.7 ± 3.4 % (10 μg/ml), 8.4 ± 1.8 % (100 μg/ml), 21.7 ± 3.4 % (250 μg/ml), 41.6 ± 2.3 % (500 μg/ml), and 55.2 ± 3.3 % (1000 μg/ml). Statistically significant differences were found at the 100, 250, 500, and 1000 μg/ml concentrations for the U87 cell lines (p values of <0.001) and at the 250, 500, and 1000 μg/ml concentrations for the T98 cell lines (p values of <0.001). IC_50_ was found at concentrations >652.78 μg/ml for the T98 cell line and >808.82 μg/ml for the U87 cell line.

For OXC, the growth inhibition for the T98G cell line for each concentration was 17.7 ± 4.1 % (2.5 μg/ml), 21.1 ± 3.6 % (5 μg/ml), 53.6 ± 14.2 % (10 μg/ml), 82.2 ± 2.3 % (20 μg/ml), and 85.0 ± 2.3 % (40 μg/ml). For U-87 MG it was 8.0 ± 11.8 % (2.5 μg/ml), 11.7 ± 5.3 % (5 μg/ml), 42.1 ± 17.6 % (10 μg/ml), 75.7 ± 4.7 % (20 μg/ml), and 89.0 ± 1.8 % (40 μg/ml). Statistically significant differences were found at the 10, 20, and 40 μg/ml concentrations for the U87 and T98 cell lines (p values of <0.001). IC_50_ was found at concentrations >9.45 μg/ml for the T98 cell line and >12.35 μg/ml for the U87 cell line.

### Overview of cytotoxicity/cell viability

As seen in Fig. 2, the only VPA and OXC in the T98 cell line and VPA, TMZ, and OXC in the U87 cell line reached over 50 % growth inhibition. In the T98 cell line, no compounds showed growth enhancement, 6 compounds (R-(−)-MHC, S-(+)-MHC, DEX, LEV, ESX, TGB) showed less than 10 % growth inhibition, and 9 compounds (VBT, PRM, GBP, TPM, LTG, PHT, TMZ, CBZ, MgSO_4_) showed 10–35 % growth inhibition. In the U87 cell line, 2 compounds showed growth enhancement (VBT 7 %, LEV 3 %), 4 compounds (ESX, DEX, TGB, PRM) showed less than 10 % growth inhibition, and 8 compounds (LGT, GBP, PHT, MgSO_4_, TPM, CBZ, R-(−)-MHC, S-(+)-MHC) showed 10–35 % growth inhibition. ESX and DEX also showed slight growth enhancement at high drug concentrations.

### Cell cycle analysis

**Fig. 3:**
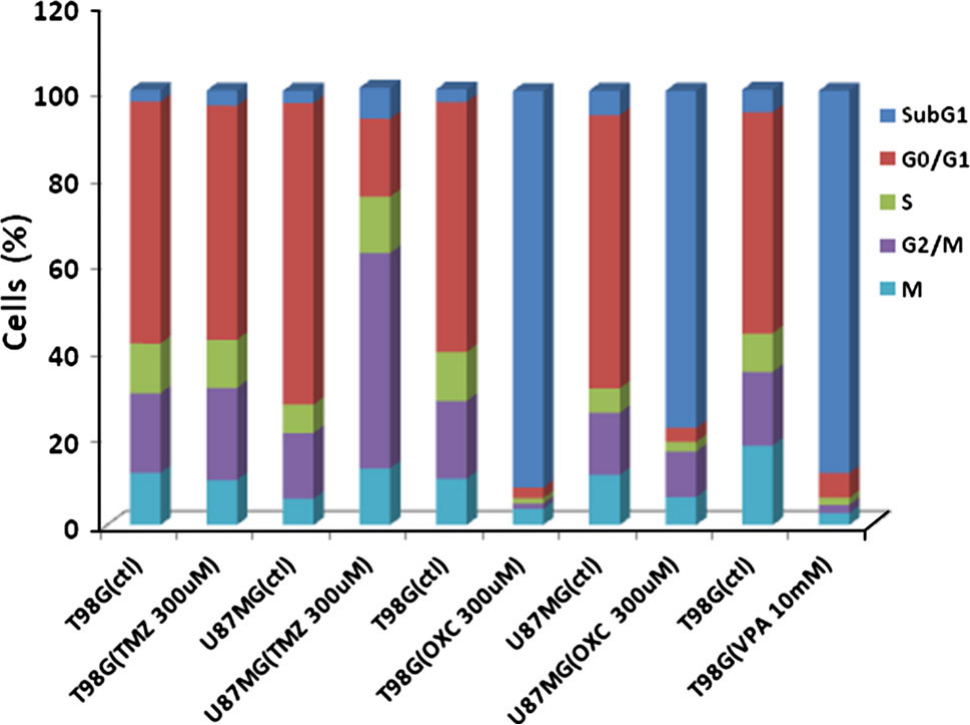
Effect of TMZ, OXC, and VPA on cell cycle in two glioma cell lines

Cell cycle analysis was performed for TMZ, OXC, and VPA. The drug concentrations used for cell cycle analysis were 300 μM for TMZ (~60 μg/ml) and OXC (~80 μg/ml), and 10 mM for VPA (~1500 μg/ml). Flow cytometry was performed and the results given in Fig. 3.

TMZ was shown to have minimal effect on the T98 cell line, but induced G2/M arrest in the U87 cell line and increased SubG1 indicating apoptosis. OXC was shown to have greatly increased the SubG1 population in both T98 (91.4 %) and U87 (77.1 %). VPA was also shown to greatly increase the SubG1 population in T98 (88.1 %). Preliminary experiments using lower concentrations and shorter incubation times for VPA and OXC indicate a possibility of G2/M arrest at shorter incubation times and similar patterns in lower concentrations as long as IC_50_ is reached.

## Discussion

In this study, we investigated three main areas, the effect of VPA alone on cancer cell growth, the concentration of VPA needed to affect cell growth, and the effects of various AEDs on cell growth.

### The effects of valproic acid

Multiple studies have shown that the use of VPA in GBM results in cell growth inhibition in both the U-87 MG and the T98G cell lines. The drug concentration doses in these studies ranged from ~35 to ~1500 μg/ml. The data from these studies indicated that at ~500 μg/ml, a significant level of growth inhibition, approximately 40–60 %, was reached [39–41].

While the results of our study did find significant growth inhibition and apoptosis caused by VPA alone, the IC_50_ was found at concentrations that would be considered toxic when used in patients. The IC_50_ calculated for both cell lines were higher than that reported by others and far exceed the normal serum concentrations levels for the treatment of seizures (50–100 ug/ml). At therapeutic concentrations, VPA alone showed less than 20 % growth inhibition. This indicates that VPA alone does not provide significant anti-cancer effects and the effects seen in our previous study on VPA in patients may be influenced by the fact that VPA was used in combination with TMZ, the chemotherapy of choice, or possibly radiation therapy (RT).

### The effects of other AEDs

Of these other compounds used in this study, only OXC was shown to have significant inhibitory effects at what would be considered therapeutic levels for the treatment of epilepsy. The results for OXC showed that OXC was effective in inhibiting cell growth, was able to attain IC_50_, and induced possible G2M arrest and apoptosis. This information is similar to another study that also showed that OXC had possible anti-cancer effects in other cell lines though the concentrations used in that study far exceeded what would be considered therapeutic for epilepsy [42]. Further study is required to determine the exact mechanism behind the inhibitory effects of OXC. Due to the fact that OXC is a prodrug, the metabolites (R-(−)-MHC and S-(+)-MHC were also assessed for cell growth inhibition. The metabolites did not reach 50 % growth inhibition even at a concentration double the maximum accepted therapeutic plasma level for OXC metabolites. This discovery indicates that since OXC is quickly metabolized into R-(−)-MHC and S-(+)-MHC when OXC is taken orally, OXC may need a local delivery system to provide anti-cancer effects and bypass metabolism.

None of the other epileptics used in this study reached IC_50_. MgSO_4_, PHT, CBZ, LTG, GBP, PRM, TGB and TPM showed overall growth inhibition in both cell lines. Even though CBZ and OXC have similar chemical structures, CBZ did not reach IC_50_ in our study and another study has shown that they are distinctly different drugs in their mode of action, metabolism, efficacy, and tolerability [43]. DEX and ESX showed slight growth inhibition in the T98 cell line, but growth enhancement at higher concentrations in the U87 cell line. LEV and VBT showed growth inhibition in the T98 cell line and growth enhancement in the U87 cell line at all concentrations. While the growth enhancement effects of ESX, LEV, and VBT failed to reach significance, the possible effects of these compounds should be taken under consideration when choosing epileptic therapy in patients with GBM.

### Impact on clinical practice

While the survey of antiepileptic compounds was performed on glioblastoma cell lines and not in actual patients, there are still some aspects of this study that can be of benefit when considering the use of antiepileptics in the treatment of GBM patients.

While toxic doses of valproic acid are required to reach significant growth inhibition in this study, our previous study did find merit in the use of VPA in conjunction with standard therapy [25]. This finding also matches other studies that have found that VPA can be of benefit in some cancer patients though not in all cancer types [44–47]. While none of the current studies found that VPA alone improved outlook for cancer patients, VPA in conjunction with other therapies was found to possibly have benefit in some cancer types [25, 44–47]. While oral VPA monotherapy is impractical in GBM, local treatment using a wafer implanted after surgery may allow for higher local saturation to reach the needed concentration.

For oxcarbazepine, no clinical studies in patients have been reported. While oxcarbazepine did reach significant growth inhibition, which matches the information found by another study, it is a prodrug that is quickly metabolized [42]. While the parent drug, oxcarbazepine, has significant inhibitory effects, the metabolites of oxcarbazepine, licarbazepine and eslicarbazepine, did not reach significant growth inhibition indicating that orally given oxcarbazepine may not be clinically useful in treating GBM. The effectiveness of oxcarbazepine does indicate that it may be useful as a local treatment in the form of wafer implanted post surgery. Additional studies will be performed to explore this possibility.

Another important aspect of this study is the finding that some antiepileptics may have slight growth enhancement effects in GBM. While the growth enhancement was not significant, it is still important to note that the antiepileptics studied fell into two basic groups. The first group, consisting of VPA, OXC, R-(−)-MHC, S-(+)-MHC, MgSO_4_, PHT, CBZ, LTG, GBP, PRM, TGB and TPM, show growth inhibition of GBM cells indicating that the use of these compounds for the treatment of epilepsy in GBM patients is safe and has no negative impact on GBM patients. The second group, consisting of ESX, LEV, and VBT, however, showed minor growth enhancement. This finding, while not reaching significance, does call into question if these compounds should be avoided when deciding on prophylactic antiepileptic therapy or choosing antiepileptic therapy for newly diagnosed patients with GBM. With the multitude of options available for antiepileptic treatment, it may be useful to consider choosing antiepileptic compounds that demonstrate no growth enhancement at all over those that showed even slight growth enhancement.

## Conclusion

Our results showed that TMZ, valproic acid, and oxcarbazepine significantly inhibited glioblastoma cell growth and that VPA and OXC may induce apoptosis or G2M arrest in glioblastoma cell lines. It was also demonstrated that OXC metabolites did not impact cell growth, therapeutic levels of VPA were not effective, and that toxic levels of VPA are needed to impact cell growth. These findings indicate that while VPA and OXC may not be useful as anti-cancer therapy when used as treatment for epilepsy, it is possible that both compounds may have efficacy when used as local treatment in the form of a wafer or local implant to bypass the issues of toxicity and metabolism. The other antiepileptic drugs screened did not show significant growth inhibition or enhancement, though the compounds that enhance cell growth may need further consideration before use in GBM patients. Future studies are needed to examine the effectiveness of AEDs when given in combination with standard GBM therapy, to define the mechanism behind OXC’s effectiveness, and to explore the possibility of using VPA or OXC as local treatment. Furthering our understanding of AEDs used in the treatment of GBM patients can lead to the development of better clinical therapy guidelines.
